# Exploiting the dynamics of hyperthermia-enhanced delivery of thermosensitive liposomal doxorubicin to solid tumors

**DOI:** 10.1080/10717544.2026.2670077

**Published:** 2026-05-10

**Authors:** Pouya Namakshenas, Johannes Crezee, H. Petra Kok

**Affiliations:** a Department of Radiation Oncology, Amsterdam UMC Location University of Amsterdam, Amsterdam, The Netherlands; b Cancer Center Amsterdam, Cancer Biology and Immunology, Amsterdam, The Netherlands; c Cancer Center Amsterdam, Treatment and Quality of Life, Amsterdam, The Netherlands

**Keywords:** Thermosensitive liposomes (TSLs), hyperthermia, intravascular drug release, drug delivery, computational modeling

## Abstract

Thermosensitive liposomal (TSL) drug delivery with intravascular release under hyperthermia is a promising approach for chemotherapy of solid tumors, where the hyperthermia schedule strongly influences delivery efficacy. This study uses mathematical modeling to evaluate these effects. A compartmental modeling approach was used to simulate TSL-encapsulated doxorubicin (DOX) delivery. The model was calibrated and validated against published *in vivo* data from murine tumor models. Key variables included hyperthermia timing relative to TSL-DOX administration (0–60 min), duration (15–90 min), and heating pattern (continuous vs. fractional). Tumor cells exhibiting multidrug resistance (MDR), based on uptake characteristics of non-small cell lung cancer (NSCLC) and breast cancer cells, were modeled by varying cellular efflux rates. Initiating hyperthermia at peak plasma TSL levels increased the maximum intracellular DOX concentration by up to twofold compared with a 60-min delay. Tumor models characterized by NSCLC-like uptake were less responsive to prolonged hyperthermia than MCF-7 and MDA-468 breast cancer cells, showing minimal additional intracellular accumulation beyond 60 min. Low-MDR tumor models exhibited greater hyperthermia-enhanced uptake than high-MDR models. Prolonged hyperthermia increased systemic exposure to free DOX; however, the relative enhancement in tumor exposure exceeded that in systemic plasma. Continuous hyperthermia yielded a 20% higher intracellular DOX concentration after 60 min compared with a fractional schedule (4 × 15 min with 15-min cool-down intervals). For optimal delivery, hyperthermia in the stationary phase is most effective when synchronized with peak plasma TSL-DOX levels. Hyperthermia duration may require cancer-type–specific adjustment. These findings provide a mechanistic basis to inform hyperthermia protocol design.

## Introduction

Poor delivery of conventional systemic chemotherapy to solid tumors, combined with high systemic toxicity, has driven the development of nanoparticle-based targeted drug delivery systems. Thermosensitive liposomal (TSL) delivery is one such nanoparticle-based system, designed to release the majority of its drug cargo at the tumor site under mild hyperthermia (typically between 39 °C and 43 °C) (Koning et al. 2010; Chaudhry et al. 2022; Haemmerich et al. 2023). The efficacy of TSL-based drug delivery depends on the liposome formulation, which determines its plasma stability and drug release rate, both of which influence the system's pharmacokinetic behavior (Kneidl et al. 2014). Depending on the release site, TSL-based delivery systems are generally classified as either extravascular (TSL-e) or intravascular (TSL-i). In the TSL-e system, drug delivery relies on passive transport by exploiting the enhanced permeability and retention (EPR) effect of tumors (Namakshenas and Mojra 2023; Regenold et al. 2023), with hyperthermia typically applied 24 h after administration (Gasselhuber et al. 2012). However, the reliability of the EPR effect in human tumors has since been questioned (Danhier 2016; Sindhwani et al. 2020; Chaudhry et al. 2022; Regenold et al. 2023; Cheng et al. 2023). In contrast, TSL-i, analogous to the infusion of chemotherapeutics into tumor-supplying vessels, releases the drug directly within the vasculature, from which it is then extracted into the tumor tissue by diffusion. In this delivery system, hyperthermia is recommended immediately after TSL administration (Haemmerich et al. 2023). Fast-releasing TSL-i systems, engineered to release over 80% of their drug payload within seconds under hyperthermic conditions, have demonstrated significantly higher tumor drug accumulation compared to slower-releasing systems and conventional chemotherapy (Gasselhuber et al. 2012; Li et al. 2013; Ten Hagen et al. 2021). This was shown in preclinical studies using murine Lewis lung carcinoma tumors with fluorescent dye-loaded TSLs (Ten Hagen et al. 2021), as well as in a rat soft-tissue sarcoma model treated with an irinotecan-loaded DPPG2-based TSL formulation (Wedmann et al. 2025). A 15-fold increase in doxorubicin (DOX) delivery was also reported for the DPPG_2_-TSL under HIFU-induced hyperthermia in porcine models, compared with conventional non-liposomal DOX (Sebeke et al. 2022). In these models, rapid intravascular release led to markedly enhanced drug bioavailability. Furthermore, early results from a Phase I clinical trial of THE001 (U S National Library of Medicine 2023; Thermosome GmbH 2025), a DPPG_2_-based TSL formulation of DOX, in patients with locally advanced or metastatic soft tissue sarcoma demonstrate that THE001 is well tolerated in combination with regional hyperthermia at a dose of 40 mg/m^2^, with preliminary evidence of clinical activity and local disease control.

The extraction fraction, which describes how effectively a drug crosses the vasculature into the tumor's extravascular extracellular space (EES), is governed by physiological parameters such as vascular density, vascular permeability, and blood perfusion rate (Li et al. 2013; Stylianopoulos et al. 2018). An increased ratio of the permeability-surface area product of the tumor vasculature to the perfusion rate leads to a higher extraction fraction and, consequently, enhances the potential for effective drug delivery (Ten Hagen et al. 2021). Furthermore, assuming a constant extraction fraction, a shorter release time from TSLs relative to the tumor transit time, which is the time required for plasma to travel from the arterial supply to the venous end, leads to greater drug bioavailability (Ten Hagen et al. 2021). While transport dynamics during hyperthermia-triggered release of TSL-i systems in relation to tumor physiological parameters have been well studied (Asemani and Haemmerich 2017; Ten Hagen et al. 2021; Haemmerich et al. 2023), the influence of hyperthermia variables, such as the timing between intravascular TSL administration and heating, as well as the duration and pattern of hyperthermia, remains underexplored in determining therapeutic efficacy, particularly given the variability in drug uptake rates among cancer cells.

Following administration, the concentration of TSLs in the bloodstream reaches a peak and then declines over time. If hyperthermia is applied too late, reduced plasma levels of intact TSLs may limit drug release at the tumor site. Supporting this concern, preclinical studies (Ramajayam et al. 2021) have shown that reduced availability of circulating DOX-loaded TSLs is associated with decreased tumor fluorescence, consistent with impaired drug accumulation in the tumor. These observations underscore the need to investigate how the interval between TSL administration and hyperthermia initiation affects drug bioavailability and intratumoral delivery.

Hyperthermia duration is an additional parameter that significantly affects drug accumulation within tumor tissue. In an *in vivo* study, Motamarry et al. (2019) showed that increasing the duration of hyperthermia from 15 to 60 min approximately doubled the concentration of DOX in Lewis lung carcinoma tumors in mice when using a fast-releasing TSL-i system. The extent of drug uptake from the tumor EES into tumor cells varies across different cancer cell types and is regulated by factors such as binding rate, saturation kinetics for chemodrug transport, and efflux rate (El-Kareh and Secomb 2005). Cellular resistance to chemotherapeutic agents is often associated with elevated efflux activity, commonly referred to as the multidrug resistance (MDR) phenotype (Eckford and Sharom 2009; Gasselhuber et al. 2012; Emran et al. 2022). As a result, the effect of hyperthermia duration on intracellular drug concentration may depend on the degree of drug resistance exhibited by tumor cells. If overlooked, this factor can hinder attainment of the desired cytotoxic dose, as it governs intracellular drug levels in relation to the bioavailable drug concentration within the tumor EES. Through mathematical modeling, the interplay between hyperthermia parameters and cellular uptake can be systematically investigated.

Mathematical modeling in cancer drug delivery provides an effective framework for evaluating treatment efficacy as a function of key influencing parameters (Bhandari et al. 2024; Namakshenas et al. 2025; 2025; Munn and Jain 2025). Through the simulation of drug kinetics, from vascular administration to drug distribution in virtual tumor environments under varying conditions, this modeling approach enables efficient systematic treatment optimization while avoiding the cost, time, and ethical concerns associated with extensive animal experimentation. Gasselhuber et al. (2012) used a pharmacokinetic model based on mouse data to propose an optimized drug release time aimed at maximizing intracellular concentrations for different liposomal formulations, including Stealth-DOX (PEGylated liposomes engineered for prolonged circulation and passive tumor accumulation via the EPR effect, rather than externally triggered release), TSL-encapsulated DOX (TSL-DOX) in both TSL-e and TSL-i forms. Their results showed that, in MDR cells with high efflux activity, the optimal drug release time must be significantly shorter. For example, the optimal release time for Stealth-DOX decreased from 45 min in drug-sensitive cells to just 4 min in MDR cells, exhibiting a 10-fold increase in efflux rate. Ten Hagen et al. (2021) developed a computational model for a TSL-i-based drug delivery system in which tumor transport parameters were quantified using intravital microscopy imaging of rodent tumor segments, with the fluorescent dye carboxyfluorescein (CF) used as the agent. The model described the dynamics of the dye from systemic administration of TSL-encapsulated CF to its accumulation in the tumor's EES. Using a computational model, Ramajayam et al. (2021) highlighted the superior efficacy of localized hyperthermia compared to large-volume hyperthermia in enhancing drug delivery.

The present study aims to investigate the impact of three key hyperthermia-related factors in the TSL-i drug delivery system: (1) the interval between TSL-DOX administration and the onset of hyperthermia, (2) the duration of hyperthermia exposure, and (3) the hyperthermia pattern (continuous: 1 × 60 min; fractional: 2 × 30 min, 4 × 15 min, and 6 × 10 min). In addition, the effect of the MDR phenotype, represented by an increased efflux rate, was examined using DOX uptake characteristics calibrated from *in vitro* data on breast cancer and non-small cell lung cancer (NSCLC). To address these aims, we employed a multi-compartment mathematical model and validated it against published *in vivo* data by comparing predicted and measured DOX uptake in tumors. The model is formulated to investigate drug-release and transport dynamics under controlled hyperthermic exposure conditions. It does not explicitly simulate heat transfer or spatial temperature distributions, but instead isolates the pharmacokinetic consequences of different hyperthermia schedules once the target temperature range is achieved. Antitumor efficacy was then assessed based on the maximum intracellular drug concentration, a metric shown to correlate strongly with DOX-induced cancer cell death *in vitro* (El-Kareh and Secomb 2005). In parallel with tumor efficacy metrics, systemic exposure to free DOX was quantified under prolonged hyperthermia schedules to evaluate trade-offs associated with enhanced intravascular drug release.

## Materials and methods

In this section, we outline the methodology used to simulate a multi-compartment mathematical model for the TSL-i drug delivery system. First, we present the model equations, followed by the validation approach using *in vivo* data from the literature and the evaluation metrics used to assess treatment efficacy. Finally, we provide a detailed description of the hyperthermia setups.

### Mathematical modeling of TSLs-based drug delivery

**Figure 1.**
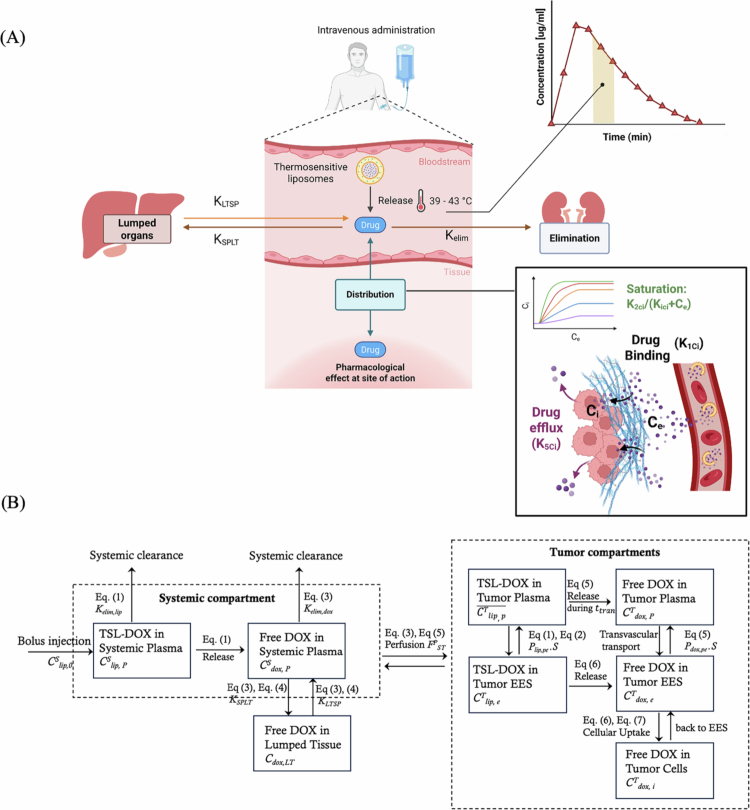
(A) Schematic of the TSL-i drug delivery system and the associated kinetic parameters used in the mathematical model. Upon administration, a fraction of the encapsulated drug is released under normothermic conditions, while the majority is released in the tumor vasculature under mild hyperthermia (39 °C–43 °C). The released free drug in the systemic plasma, with release from TSLs described by $\mathrm{Rl}s_{S}(t)$, undergoes elimination ($k_{\mathrm{elim}}$), distribution to the lumped tissue ($k_{\mathrm{SPLT}}$), and return from the lumped tissue to the systemic plasma ($k_{\mathrm{LTSP}}$). Within the tumor, free drug extravasates from the vasculature into the tumor EES ($C_{\mathrm{drug},e}^{T}$) and is taken up by tumor cells, modeled as a combination of linear uptake ($K_{1\mathrm{ci}}$), saturable uptake $\left(K_{2\mathrm{ci}}/(K_{\mathrm{ci}}+C_{\mathrm{drug},e}^{T})\right)$, and efflux from cells back into the EES ($K_{5\mathrm{ci}}$). Various hyperthermia schedules were examined in this study. This figure was created with BioRender.com. (B) Compartmental model structure showing the mathematical framework with equation references.

The model consists of multiple compartments, including systemic plasma, tumor plasma, lumped tissue (i.e. the remaining body tissues), the tumor EES, and the intracellular space of tumor cells. The transport of TSL-DOX is modeled through key processes: systemic exchange with lumped tissue, systemic elimination, perfusion-driven transport from systemic plasma to tumor plasma, temperature-triggered drug release, transvascular transport into the tumor EES, and subsequent cellular uptake (Figure 1). To describe these dynamics, two coupled sets of equations are solved: one describing the pharmacokinetics of TSL-DOX, and the other describing free DOX. TSL-DOX pharmacokinetics are governed by the following equations (Gasselhuber et al. 2012; Asemani and Haemmerich 2017):

(1a) $$\frac{dC_{\mathrm{lip},p}^{S}}{\mathrm{dt}}=-\underset{\mathrm{TSL}\;\mathrm{release}\;\mathrm{in systemic}\;\mathrm{plasma}\;\mathrm{at}\;37{}^{\circ}C}{\underbrace{\mathrm{Rl}s_{S}(t)\;\cdot \;C_{\mathrm{lip},p}^{S}}}-\underset{\mathrm{TSL}\;\mathrm{release}\;\mathrm{during}\;\mathrm{tumor}\;\mathrm{transit}}{\underbrace{F_{p}^{\mathrm{ST}}\;\cdot \;\left(V_{p}^{T}/V_{p}^{S}\;.\;v_{p}\right)\;\cdot \;C_{\mathrm{lip},p}^{S}(t)\;\cdot \;f_{\mathrm{loss}}(t)}}-\underset{\mathrm{Systemic}\;\mathrm{clearance}\;\mathrm{of}\;\mathrm{TSL}}{\underbrace{k_{\mathrm{elim},\mathrm{lip}}\;\cdot \;C_{\mathrm{lip},p}^{S}}}-\underset{\mathrm{Transvascular}\;\mathrm{transport}}{\underbrace{P_{pe}^{lip}\;\cdot \;S_{pe,vas}\;\cdot \;(V_{p}^{T}/V_{p}^{S}\;.\;v_{p})}}\;\cdot \;\left(\underset{\mathrm{Avg}.\mathrm{TSL}\;\mathrm{conc}\;\mathrm{in}\;\mathrm{the}\;\mathrm{tumor}\;\mathrm{plasma}}{\underbrace{\bar{C_{lip,p}^{T}}(t)}}-\underset{\mathrm{TSL}\;\mathrm{in}\;\mathrm{tumor}\;\mathrm{EES}}{\underbrace{C_{lip,e}^{T}}}\right),$$

(1b) $$f_{\mathrm{loss}}(t)=\left\{ \begin{array}{ll} 1-\frac{1-\alpha_{37}+\alpha_{37}\cdot e^{-R_{37}\cdot (t-t_{\mathrm{tran}})}}{1-\alpha_{37}+\alpha_{37}\cdot e^{-R_{37}\cdot t}}, & \mathrm{if}\;t<t_{\mathrm{heat},\mathrm{start}}\;\mathrm{or}\;t\geq t_{\mathrm{heat},\mathrm{end}}\\ \alpha_{42}\;(1-e^{-R_{42}\cdot t_{\mathrm{tran}}}), & \mathrm{if}{\;t}_{\mathrm{heat},\mathrm{start}}\leq t<t_{\mathrm{heat},\mathrm{end}} \end{array}\right.,$$

(1c) $$\bar{C_{\mathrm{lip},p}^{T}}(t)=C_{\mathrm{lip},p}^{S}(t)\;\left[1-\alpha +\frac{\alpha }{R\cdot t_{\mathrm{tran}}}\left(1-e^{-R\cdot t_{\mathrm{tran}}}\right)\right],$$

(2a) $$\frac{dC_{\mathrm{lip},e}^{T}}{\mathrm{dt}}=-\underset{\mathrm{Temperature}-\mathrm{triggered}\;\mathrm{release}\;\mathrm{of}\;\mathrm{TSL}\;\mathrm{in}\;\mathrm{tumor}}{\underbrace{\mathrm{Rl}s_{T,e}(t)\cdot C_{\mathrm{lip},e}^{T}}}+\underset{\mathrm{Transvascular}\;\mathrm{transport}\;\mathrm{of}\;\mathrm{TSL}}{\underbrace{(P_{\mathrm{pe}}^{\mathrm{lip}}\cdot S_{\mathrm{pe},\mathrm{vas}}/v_{e})\cdot \left(\bar{C_{\mathrm{lip},p}^{T}}(t)-C_{\mathrm{lip},e}^{T}\right)}},$$

(2b) $${\mathrm{Rls}}_{T}(t)=\frac{\alpha_{\theta }\cdot R_{\theta }\cdot e^{-R_{\theta }\cdot t}}{1-\alpha_{\theta }+\alpha_{\theta }\cdot e^{-R_{\theta }\cdot t}},\;\;\;\;\theta \;=\;37\;{}^{\circ}C\;or\;42\;{}^{\circ}C$$

where in Equations (1–2), $C_{\mathrm{lip},p}^{S}$ and $C_{\mathrm{lip},e}^{T}$ [μg/mL] are the liposomal DOX concentrations in systemic plasma and tumor EES, respectively. In this framework, tumor plasma is not treated as a distinct compartment for liposomal DOX; rather, its effects are incorporated into the dynamics of adjacent compartments (Asemani and Haemmerich 2017). Specifically, as TSLs transit through the tumor, a fraction ($f_{\mathrm{loss}}$, (1b)) is released due to hyperthermia. This release is represented as a sink term in the systemic plasma concentration of liposomal DOX and as a corresponding source term for free DOX in the tumor plasma. The average liposomal DOX concentration in tumor plasma (1c) was modeled assuming plug-flow transit of systemic plasma through the tumor vasculature combined with temperature-dependent drug release kinetics. The transit time through the tumor, $t_{\mathrm{tran}}$, is defined as the ratio of tumor plasma volume fraction to tumor blood perfusion rate: $t_{\mathrm{tran}}=\frac{v_{p}}{F_{\mathrm{pST}}}$ (Asemani and Haemmerich 2017; Ten Hagen et al. 2021). The initial dose of TSL-DOX was set to 0.1 mg, equivalent to the *in vivo* dose of 5 mg/kg administered to mice (Motamarry et al. 2019) with an average body weight of 20 g. The compartmental release rate Rls(t) in (2b) models the dynamic release of TSLs while accounting for incomplete release. The characteristic release rate (*R*), is defined as the inverse of the characteristic release time, τ, where τ denotes the time required to achieve approximately 63% of the maximum release (α) for a given temperature and TSL formulation. The release kinetics of TSLs, as well as their elimination rate, depend on the specific TSL formulation, which in turn affects the amount of free DOX. The pharmacokinetics of free DOX can be described as follows (Gasselhuber et al. 2012; Asemani and Haemmerich 2017):

(3) $$\begin{array}{l} \frac{dC_{\mathrm{dox},p}^{S}}{\mathrm{dt}}=-\underset{\mathrm{Exchange}\;\mathrm{with}\;\mathrm{tumor}\;\mathrm{plasma}}{\underbrace{(V_{p}^{T}/V_{\mathrm{dist}}^{S})\;\cdot \;(F_{p}^{\mathrm{ST}}/v_{p}^{T})\;\cdot \;\left(C_{\mathrm{dox},p}^{S}-C_{\mathrm{dox},p}^{T}\right)}}-\underset{\mathrm{Elimination}}{\underbrace{k_{\mathrm{elim},\mathrm{dox}}\;\cdot \;C_{\mathrm{dox},p}^{S}}}\\ +\underset{\mathrm{Return}\;\mathrm{from}\;\mathrm{lumped}\;\mathrm{tissue}}{\underbrace{k_{\mathrm{LTSP}}\;\cdot \;C_{\mathrm{dox},\mathrm{LT}}}}-\underset{\mathrm{Distribution}\;\mathrm{to}\;\mathrm{lumped}\;\mathrm{tissue}}{\underbrace{k_{\mathrm{SPLT}}\;\cdot \;C_{\mathrm{dox},p}^{S}}},\\ \;+\underset{\mathrm{Released}\;\mathrm{drug}\;\mathrm{from}\;\mathrm{TSLs}\;\mathrm{in}\mathrm{to}\;\mathrm{plasma}\;\mathrm{at}\;37\;{}^{\circ}C}{\underbrace{\mathrm{Rl}s_{S}(t)\;\cdot \;C_{\mathrm{lip},p}^{S}\;\cdot \;\frac{V_{p}^{S}}{V_{\mathrm{dist}}^{S}}}} \end{array}$$

(4) $$\frac{dC_{\mathrm{dox},\mathrm{LT}}}{\mathrm{dt}}=-\underset{\mathrm{Return}\;\mathrm{from}\;\mathrm{lumped}\;\mathrm{tissue}}{\underbrace{k_{\mathrm{LTSP}}\;\cdot \;C_{\mathrm{dox},\mathrm{LT}}}}+\underset{\mathrm{Distribution}\;\mathrm{to}\;\mathrm{lumped}\;\mathrm{tissue}}{\underbrace{k_{\mathrm{SPLT}}\;\cdot \;C_{\mathrm{dox},p}^{S}}},$$

(5) $$\begin{array}{l} \frac{dC_{\mathrm{dox},p}^{T}}{\mathrm{dt}}=\underset{\mathrm{Exchange with tumor plasma}}{\underbrace{(F_{p}^{\mathrm{ST}}/v_{p})\;\cdot \;\left(C_{\mathrm{dox},p}^{S}-C_{\mathrm{dox},p}^{T}\right)}}\\ -\underset{\mathrm{Transvascular transport}}{\underbrace{P_{\mathrm{pe}}^{\mathrm{dox}}\;\cdot \;S_{\mathrm{pe},\mathrm{vas}}/v_{p}}}\;\cdot \;\left(\underset{\mathrm{Drug in tumor plasma}}{\underbrace{C_{\mathrm{dox},p}^{T}}}-\underset{\mathrm{Drug in tumor EES}}{\underbrace{C_{\mathrm{dox},e}^{T}}}\right)\\ +\underset{\mathrm{Released drug within vasculature during tumor transit}}{\underbrace{\left(F_{p}^{\mathrm{ST}}/v_{p})\;\cdot \;C_{\mathrm{lip},p}^{S}(t)\;\cdot \;f_{\mathrm{loss}}(t\right)}} \end{array}$$

(6a) $$\begin{array}{l} \frac{dC_{dox,e}^{T}}{\mathrm{dt}}=\underset{\mathrm{Transvascular}\;\mathrm{transport}}{\underbrace{P_{\mathrm{pe}}^{dox}\;\cdot \;S_{\mathrm{pe},\mathrm{vas}}/v_{e}}}\;\cdot \;\left(\underset{\mathrm{Drug}\;\mathrm{in}\;\mathrm{tumor}\;\mathrm{plasma}}{\underbrace{C_{dox,p}^{T}}}-\underset{\mathrm{Drug}\;\mathrm{in}\;\mathrm{tumor}\;\mathrm{EES}}{\underbrace{C_{\mathrm{dox},e}^{T}}}\right)\\ -\underset{\mathrm{Cellular}\;\mathrm{uptake}\;\mathrm{of}\;\mathrm{DOX}}{\underbrace{v_{i}/v_{e}\;\cdot \;\mathrm{uptake}}}+\underset{\mathrm{Released}\;\mathrm{drug}\;\mathrm{from}\;\mathrm{TSL}\;\mathrm{in}\;\mathrm{tumor}\;\mathrm{EES}}{\underbrace{\mathrm{Rl}s_{T,e}(t)\;\cdot \;C_{\mathrm{lip},e}^{T}}} \end{array},$$

(6b) $$\mathrm{uptake}=k_{3\mathrm{ci}}(\underset{\mathrm{Linear}\;\mathrm{term}}{\underbrace{k_{1\mathrm{ci}}c_{e}^{T}}}+\underset{\mathrm{Saturable}\;\mathrm{term}}{\underbrace{\frac{k_{2\mathrm{ci}}c_{e}^{T}}{K_{\mathrm{ici}}+c_{e}^{T}}}}-\underset{\mathrm{Efflux}\;\mathrm{term}}{\underbrace{k_{5\mathrm{ci}}c_{i}^{T}}}),$$

(7) $$\frac{dC_{\mathrm{dox},i}^{T}}{\mathrm{dt}}=\underset{\mathrm{Cellular}\;\mathrm{uptake}\;\mathrm{of}\;\mathrm{DOX}}{\underbrace{\mathrm{uptake}}}$$

where in Equations (3–7), $C_{\mathrm{dox},p}^{S}$, $C_{\mathrm{dox},\mathrm{LT}}$, $C_{\mathrm{dox},p}^{T}$, $C_{\mathrm{dox},e}^{T}$, and $C_{\mathrm{dox},i}^{T}$ [μg/mL] denote the DOX concentrations in systemic plasma, lumped tissue, tumor plasma, tumor EES, and tumor intracellular space, respectively. In Equation (6 and 7), the cellular uptake rate was modeled considering linear binding, saturation kinetics, and efflux from the intracellular space to the EES (El-Kareh and Secomb 2005). The model variables and parameters are defined in Table 1.

**Table 1.** Physiological, kinetic, and dosing parameters for the mathematical modeling of TSL-DOX delivery.

Category	Parameter	Definition	Rodent value
**Compartment volumes**			
Physiological	$V_{p}^{S}$	Systemic plasma volume	1.12 mL Ten Hagen et al. (2021)
Physiological	$V_{p,\mathrm{dist}}^{S}$	Systemic distribution volume	8.99 mL Ten Hagen et al. (2021)
Physiological	$V_{p}^{T}$	Tumor plasma volume	0.3 × 10^−^^3^ mL Ten Hagen et al. (2021)
Physiological	*v*_*p*_	Tumor plasma volume fraction	0.23 Ten Hagen et al. (2021)
Physiological	*v* *_e_*	Tumor EES volume fraction	0.28 Ten Hagen et al. (2021)
Physiological	*v* *_i_*	Tumor intracellular space volume fraction	0.49 Ten Hagen et al. (2021)
Physiological	$\rho_{\mathrm{cells}}$ ^a^	Tumor cell density	5 × 10^8^ cells/mL Del Monte (2009)
**Transport parameters**			
Physiological	$F_{p}^{\mathrm{ST}}$	Plasma flow rate from systemic to tumor	0.047 ${\mathrm{mL}}_{\mathrm{blood}}/({\mathrm{mL}}_{\mathrm{tissue}}\cdot s)$ Ten Hagen et al. (2021)
Physiological	$P_{\mathrm{pe}}^{\mathrm{dox}}\;\cdot \;S_{\mathrm{pe},\mathrm{vas}}$	DOX permeability-surface area product	39.6 h^−^^1^ Ramajayam et al. (2021)
Physiological	$P_{\mathrm{pe}}^{\mathrm{lip}}\;\cdot \;S_{\mathrm{pe},\mathrm{vas}}$	TSL permeability-surface area product	0.245 h^−^^1^ Gasselhuber et al. (2012)
Physiological	*t*_tran_	Transit time across tumor	5 s Ten Hagen et al. (2021)
**Free DOX rate constants**			
PK	$k_{\mathrm{elim},\mathrm{dox}}$	Systemic elimination rate of DOX	7.56 h^−^^1^ Ramajayam et al. (2021)
PK	$k_{\mathrm{SPLT}}$	DOX transfer rate from plasma to lumped tissue	33.8 h^−^^1^ Ramajayam et al. (2021)
PK	$k_{\mathrm{LTSP}}$	DOX transfer rate from lumped tissue to plasma	0.252 h^−^^1^ Ramajayam et al. (2021)
**Dosing parameters**			
Dosing	ID	Total DOX dose	0.1 mg Motamarry et al. (2019)
Dosing	$C_{0,\mathrm{lip},\mathrm{sys}}$	Initial TSL concentration in systemic plasma	89.3 μg/mL
**TSL-specific parameters**			
TSL-specific	$k_{\mathrm{elim},\mathrm{lip}}$	Systemic elimination rate for liposomes	0.8 h^−^^1^ Gasselhuber et al. (2012)
TSL-specific	α_37_	Fractional DOX release at 37 °C	0.2 Gasselhuber et al. (2012)
TSL-Specific	$\alpha_{42}$	Fractional DOX release at 42 °C	0.8 Gasselhuber et al. (2012)
TSL-Specific	$\tau_{37}$	Leakage time constant at 37 °C	120 s Gasselhuber et al. (2012)
TSL-Specific	$\tau_{42}$	Release time constant at 42 °C	2 s Gasselhuber et al. (2012)

^a^ The estimated tumor cell density corresponds to the expected cellularity for cancer cells with diameters of 10−16 μm (Del Monte 2009; Hao et al. 2018).

### Calibration of cellular uptake parameters

**Figure 2.**
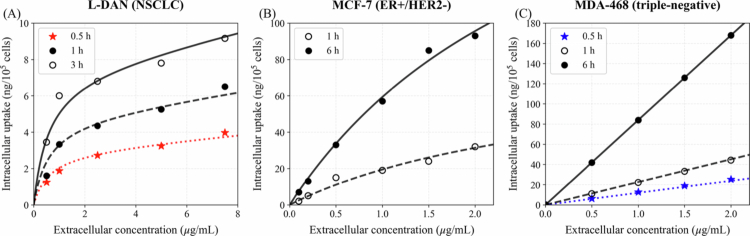
Comparison between model fits using the parameters listed in Table 2 and literature data of intracellular DOX uptake for (A) L-DAN (Kerr et al. 1986), (B) MCF-7 (Bontenbal et al. 1998), and (C) MDA-468 (Lankelma et al. 2000) cell lines. Symbols represent literature data, and lines indicate model predictions.

The parameters of the cellular pharmacokinetic uptake model described in Equation (6b) (El-Kareh and Secomb 2005) were estimated using nonlinear parameter optimization based on differential evolution-assisted least-squares minimization. Literature data from three cell lines were used: L-DAN (a type of NSCLC) (Kerr et al. 1986), MCF-7 (ER+/HER2− breast cancer) (Bontenbal et al. 1998), and MDA-MB-468 (triple-negative breast cancer) (Lankelma et al. 2000). The rate constants for DOX in L-DAN (Kerr et al. 1986) and MCF-7 (Bontenbal et al. 1998) cells were determined from *in vitro* uptake data obtained at various incubation times, whereas for MDA-MB-468 cells, the same fitting approach was applied following the two-compartment model proposed by Lankelma et al. (2000), to maintain a uniform cellular pharmacokinetic uptake model. The calibrated model parameters for each cell line are summarized in Table 2. Figure 2 shows the cellular uptake model fit to the corresponding reference data. As the intracellular uptake was reported per number of cells, it was converted using the cellular density $\rho_{\mathrm{cells}}$ to express it later in our analysis in μg/mL, consistent with the units used for the other compartments.

**Table 2.** Cellular uptake parameters for different cell lines.

Parameter	Definition	L-DAN (NSCLC) Kerr et al. (1986)	MCF-7 (ER+/HER2− breast cancer) Bontenbal et al. (1998)	MDA-468 (triple-negative breast cancer) Lankelma et al. (2000)
**Cellular uptake parameters**				
$K_{1\mathrm{ci}}$	Linear binding rate constant (ng/10^5^ cells)/(μg/mL)	0.3274	0.017	114.07
$K_{2\mathrm{ci}}$	Saturable binding constant ng/10^5^ cells	7.9109	284.46	27.44
$K_{3\mathrm{ci}}$	Uptake rate constant h^−1^	0.9599	0.334	0.2189
$K_{\mathrm{ici}}$	Half-saturation constant μg/mL	0.5861	3.15	35.22

### Model validation, data analysis and evaluation metrics

The model was validated using data from two distinct experimental setups reported in the literature for the TSL-i delivery system: one measuring drug distribution in the tumor extracellular space, and the other evaluating mean tumor drug uptake, which mainly arises from intracellular space. The release kinetics were adjusted separately for each study to match the specific TSL formulation used. The validation datasets by Ten Hagen et al. (2021) and Motamarry et al. (2019) were obtained under controlled mild hyperthermia conditions (≈42 °C), ensuring that the release threshold for TSLs was reached within the tumor region of interest. Because TSL release exhibits plateau-type kinetics above this threshold, the validation primarily captures the relationship between heating duration and tumor drug accumulation rather than spatial temperature gradients.

In the first study, Ten Hagen et al. (2021) used TSL-encapsulated CF dye as a tracer to measure its concentration in tumor segments subjected to 5 min of mild hyperthermia, employing intravital microscopy. Because this dye is not taken up by cells, the intracellular compartment was excluded from the equations, and the measured concentration was attributed entirely to the tumor EES. For validation, the initial dose was set at 8 μmol/kg, and the model parameters were adjusted based on the reported measurements.

In the second study, Motamarry et al. (2019) administered 5 mg/kg TSL-encapsulated DOX to monitor the uptake of free DOX in mice bearing subcutaneous Lewis lung carcinoma tumors in real time. The model predictions for mean tumor DOX uptake were calculated using $C_{\mathrm{mean}}=C_{i}v_{i}+C_{e}v_{e}+C_{p}v_{p}$ and were compared with experimental data obtained at 15, 30, and 60 min of hyperthermia. Additionally, to verify the amount of drug supplied to the tumor from the vasculature, the systemic TSL-DOX concentration following the post-infusion peak was compared with the corresponding experimental measurements. Since pharmacological parameter measurements were not within the scope of that study, model parameters were assigned based on *in vivo* measurements reported in Ref. (Ten Hagen et al. 2021). While the volume fractions from Ref. (Ten Hagen et al. 2021) were retained, pharmacokinetic parameters for DOX (Ramajayam et al. 2021) were used in place of those for the CF dye, to align with the drug employed in the work of Motamarry et al. (2019). Given that both studies involved murine Lewis lung carcinoma tumors, the assumption used in the model is deemed valid for comparison with the experimental data. To align with the lung tumor grown in the mice, the cellular uptake term in Equation (6b) used drug transport parameters for DOX related to NSCLC, as available in the literature (Kerr et al. 1986). The uncertainty in cellular uptake characteristics was addressed by modeling different uptake levels through variations in the efflux rate parameter, ranging from 0.2 to 5 times the base value. The resulting data are presented with uncertainty error bars to reflect this assumption.

The metric used to assess the efficiency of the drug delivery system was defined as the maximum intracellular concentration $C_{i,\;\max}$, as it represents the most relevant indicator of DOX-induced cancer cell death (El-Kareh and Secomb 2005). In addition, the area under the concentration-time curve (AUC) was also evaluated by integrating the concentration over time.

### Hyperthermia initiation schedule following TSL administration

To evaluate the impact of delaying hyperthermia application relative to the peak systemic concentration of TSL-DOX on the $C_{i,\;\max}$ of DOX, we modeled delays ranging from 0 to 60 min in 5-min intervals. Different efflux rates $K_{5\mathrm{ci}}$, ranging from 1 to 10 times the baseline value, were also parametrically varied to account for the MDR tumor cells. The cellular uptake of DOX was modeled for L-DAN (NSCLC), MCF-7 (ER+/HER2− breast cancer), and MDA-468 (triple-negative breast cancer). Results were summarized in heatmap matrices showing both the absolute $C_{i,\;\max}$ and the reduction relative to the optimal condition, with rows representing hyperthermia delay and columns representing efflux rate constants.

### Hyperthermia duration schedule

To evaluate the combined influence of hyperthermia duration and cancer cell chemoresistance on treatment efficacy, a series of simulations was conducted in which the efflux rate constant ($K_{5\mathrm{ci}}$) was varied from 1× to 10× its baseline value. Hyperthermia was applied for 15, 45, 60, or 90 min, and $C_{i,\;\max}$ was computed for each condition. For each cancer type, results were arranged in a matrix with rows denoting duration and columns denoting efflux rate constants. This matrix was visualized as a heatmap to facilitate comparison of $C_{i,\;\max}$ across scenarios. Higher $K_{5\mathrm{ci}}$ values represent stronger efflux (higher drug resistance), and longer hyperthermia durations represent extended drug release periods. Color gradients indicate the magnitude of the $C_{i,\;\max}$ (μg/mL). To further illustrate how resistance influences treatment outcome, $C_{i,\;\max}$ values were extracted across efflux rates at fixed duration and presented as line plots. Finally, time-course simulations illustrated intracellular concentration dynamics during and after heating for representative efflux rates ($K_{5\mathrm{ci}}=2$ and 10) and durations.

### Systemic exposure under different hyperthermia schedules

While tumor uptake metrics ($C_{i,\;\max}$ and AUC) describe therapeutic efficacy, systemic exposure to free DOX is a critical determinant of dose-limiting cardiotoxicity. To assess the impact of hyperthermia scheduling on systemic exposure, systemic free DOX AUC was quantified in parallel with tumor AUC for varying hyperthermia start times and durations. This analysis was conducted for both the baseline tumor size and a tumor that was 10-fold larger, while assuming identical physiological properties, to evaluate the influence of tumor size on the extent of drug release, tumor uptake, and systemic exposure. Uncertainty in tumor physiological parameters was incorporated into the analysis by assuming a 20% standard deviation.

### Hyperthermia delivery patterns: continuous and fractional

Fractional heating schedules have been proposed, in some cases, as a strategy to mitigate perfusion-related heat loss and respiratory motion-induced temperature fluctuations during clinical MRgFUS treatments (Santos et al. 2017). We examined the effect of fractional hyperthermia protocols in which the total heating duration was equivalent to sustained hyperthermia. Specifically, three fractional patterns were evaluated: (i) 2 × 30 min with 30 min of cool-down, (ii) 4 × 15 min with 15 min of cool-down, and (iii) 6 × 10 min with 5 min of cool-down between heating cycles. These protocols were compared with continuous 60-min hyperthermia, and the resulting $C_{i,\;\max}$ and AUC profiles were evaluated.

## Results

In this section, we first validate the model against *in vivo* experimental data from the literature and present the temporal profile of DOX concentration in various compartments. Next, we investigate the impact of the timing of hyperthermia relative to TSL-DOX administration on treatment efficacy. Finally, we examine how hyperthermia duration and pattern influence tumor cellular DOX concentration.

### Model validation and DOX kinetics

**Figure 3.**
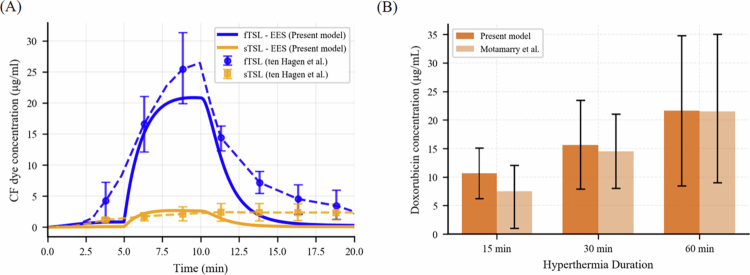
Comparison of the present model predictions with two distinct sets of experiments performed in murine Lewis lung carcinoma tumors: (A) Ten Hagen et al. (2021), where slow- and fast-release TSL-i systems encapsulating CF dye were used to measure CF concentration in the tumor EES under 5 min of hyperthermia; and (B) Motamarry et al. (2019), where a fast-release TSL-i system was applied under varying hyperthermia durations of 15, 30, and 60 min, and the mean tumor uptake of DOX was measured. The error bars in (B) indicate variation in model output resulting from changes in the efflux rate constant, which influences cellular uptake.

**Figure 4.**
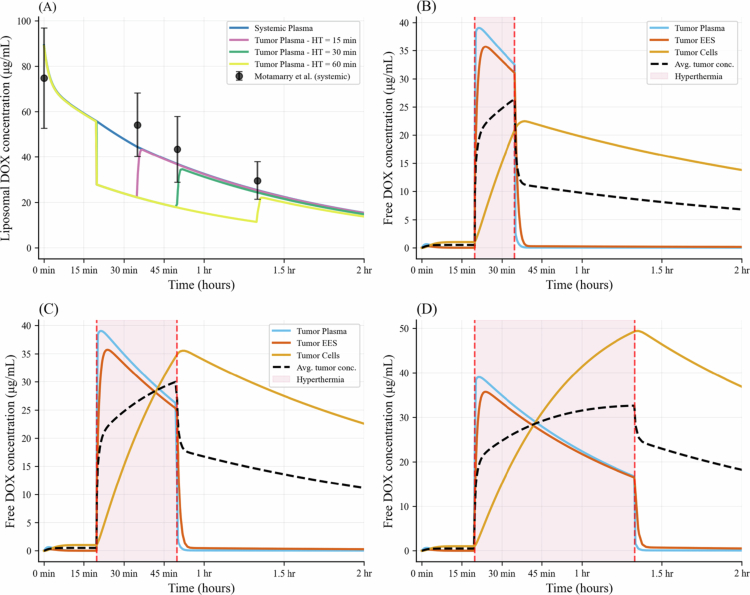
Temporal profiles of (A) TSL-Dox in systemic circulation and tumor plasma, and free Dox in tumor compartments under hyperthermia durations of (B) 15 min, (C) 30 min, and (D) 60 min, corresponding to the cases shown in Figure 3B. The systemic concentration of TSL-Dox is compared with the *in vivo* measurements reported in Ref. (Motamarry et al. 2019).

The comparison between the present model and the *in vivo* experiments by Ten Hagen et al. (2021) and Motamarry et al. (2019) is shown in Figure 3A and B, respectively. In Figure 3A, the concentration of CF dye in the tumor EES, delivered using the fast and slow TSL-i systems, is presented. The model prediction aligns with the experimental range, with some deviations that may be due to parameter uncertainty and tumor heterogeneity. In Figure 3B, both the present model and experimental data from the literature show that DOX concentration increases with longer hyperthermia duration. In particular, the model predicts mean tumor DOX concentrations of approximately 10 μg/g, 15 μg/g, and 21 μg/g for heating durations of 15, 30, and 60 min, respectively. These values are in close agreement with the corresponding experimental measurements of about 7 μg/g, 14 μg/g, and 21 μg/g, confirming the model's ability to accurately capture the observed increase in drug uptake with extended hyperthermia exposure. Figure 4 presents the model-predicted concentration of TSL-DOX in the plasma and free DOX in different compartments, corresponding to the cases shown in Figure 3B. The systemic TSL-DOX concentration in Figure 4A falls within the range reported by Motamarry et al. (2019), in which mice were maintained at a core temperature of 37 °C. Figure 4B–D shows the dynamic concentration profiles of DOX for hyperthermia durations of 15 min, 30 min, and 60 min, respectively. Following the onset of hyperthermia, tumor plasma concentrations of free DOX rose sharply, with drug subsequently diffusing into the EES and gradually accumulating within tumor cells. Intracellular concentrations reached their maximum with a short delay relative to the EES peak. Termination of heating led to a rapid fall in plasma and EES concentrations, while intracellular levels declined more slowly, consistent with sustained retention within cells. Longer heating intervals extended the period of elevated EES concentrations, thereby enhancing drug availability for uptake and resulting in higher intracellular exposure compared with shorter treatments (Figure 4B–D).

### Effect of hyperthermia initiation

**Figure 5.**
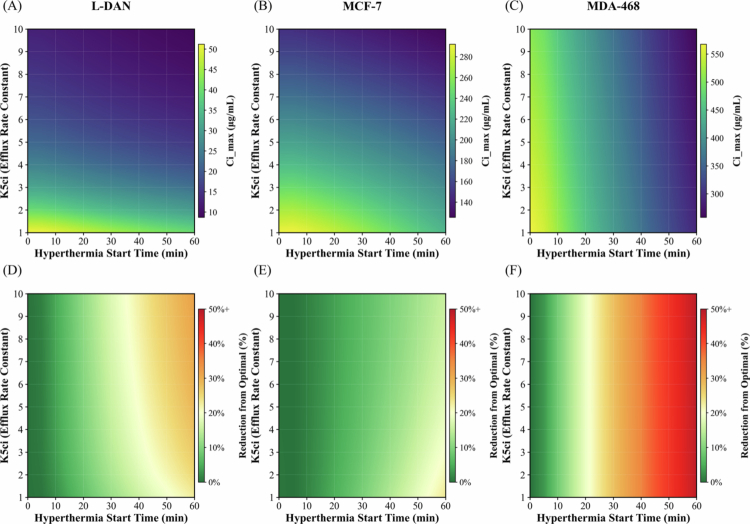
Heatmaps showing the impact of hyperthermia initiation time relative to the plasma TSL peak on $C_{i,\;\max}$, with a hyperthermia duration of 60 min. (A–C) Absolute $C_{i,\;\max}$ values and (D–F) the corresponding percentage reduction from the optimal condition, plotted as a function of efflux rate constant $K_{5\mathrm{ci}}$ (1–10) and start time (0–60 min). Panels (A) and (D) correspond to L-DAN NSCLC, (B) and (E) to MCF-7 breast cancer, and (C) and (F) to MDA-468 breast cancer.

The impact of initiating hyperthermia at different times relative to the peak systemic plasma concentration of TSL-DOX, while accounting for MDR cells, is shown in Figure 5. Figure 5A–C illustrates the absolute $C_{i,\;\max}$ values obtained for delays of up to 60 min (in 5-min intervals) during 60-min hyperthermia sessions, across a range of efflux rate constants. The corresponding percentage reductions relative to the optimal condition are shown in Figure 5D–F. In both NSCLC and breast cancer models, delaying hyperthermia decreased $C_{i,\;\max}$. Model predictions indicate that a 60-min delay in hyperthermia reduces delivery system effectiveness by up to 30% based on the uptake characteristics of L-DAN and MCF-7, and by more than 50% in MDA-468 cells.

### Impact of hyperthermia and MDR on intracellular drug accumulation

**Figure 6.**
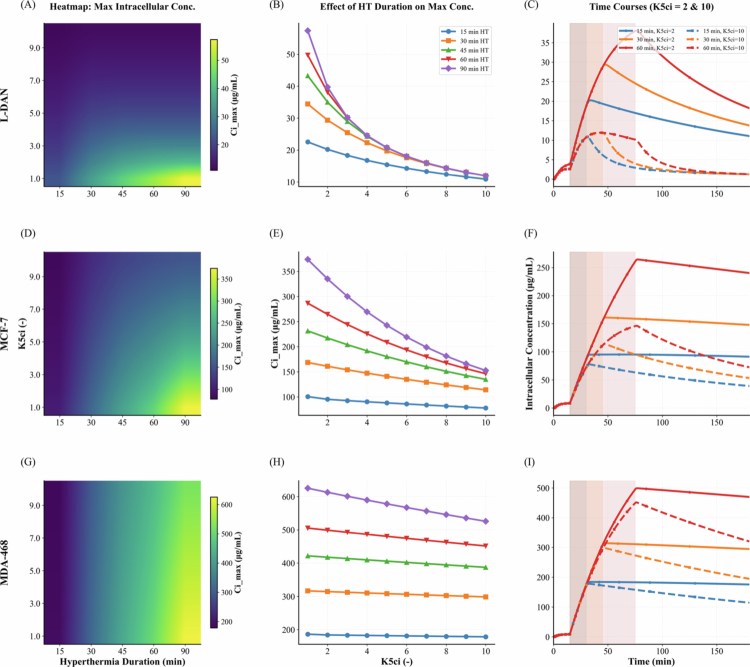
(A, D, G) Heatmaps showing the dependence of $C_{i,\;\max}$ on hyperthermia duration and efflux rate. (B, E, H) Corresponding $C_{i,\;\max}$ curves across efflux rates at selected hyperthermia durations. (C, F, I) Time-course profiles of intracellular DOX concentration for two representative efflux rates: $K_{5\mathrm{ci}}=2$ and $K_{5\mathrm{ci}}=10$. Panels (A–C) correspond to L-DAN NSCLC, (D–F) to MCF-7 breast cancer, and (G–I) to MDA-468 breast cancer.

The intracellular concentration of DOX resulting from different hyperthermia durations (15–90 min) and varying tumor cell efflux rates (*K*_5ci_ = 1–10) is shown in Figure 6. Overall, longer hyperthermia durations generally led to higher intracellular concentrations in both breast cancer and NSCLC. However, the magnitude of this effect depended on cancer type, being most pronounced based on the cellular uptake characteristics of MDA-468, followed by MCF-7, and least pronounced in L-DAN. The benefit of hyperthermia diminished substantially in MDR tumor cells with high efflux rates. For shorter heating intervals (up to 30 min for L-DAN and up to 60 min for MCF-7), intracellular concentrations increased across all efflux levels. Beyond these durations, further gains were predicted primarily in DOX-sensitive cells (low efflux rates), while concentrations plateaued in highly MDR cells. For example, in DOX-sensitive L-DAN cells ($K_{5\mathrm{ci}}=1.0$), extending hyperthermia from 15 to 30 min increased intracellular DOX from 22 to 35 μg/mL (1.6-fold). Prolonging treatment to 60 min raised it further to 50 μg/mL, a 1.4-fold increase relative to 30 min. In moderately MDR cells ($K_{5\mathrm{ci}}=5.0$), concentrations rose from 15 μg/mL at 15 min to 20 μg/mL at 30 min (1.3-fold), and only slightly to 21 μg/mL at 60 min (1.05-fold). In highly MDR cells ($K_{5\mathrm{ci}}=10.0$), $C_{i,\;\max}$ increased only marginally from 11 μg/mL at 15 min to 11.5 μg/mL at 30 min (1.05-fold), with no further gain at 60 min. Nevertheless, despite $C_{i,\;\max}$ remaining unchanged in strongly resistant cells, the AUC continued to rise with prolonged hyperthermia (Figure 6C, F, I).

**Figure 7.**
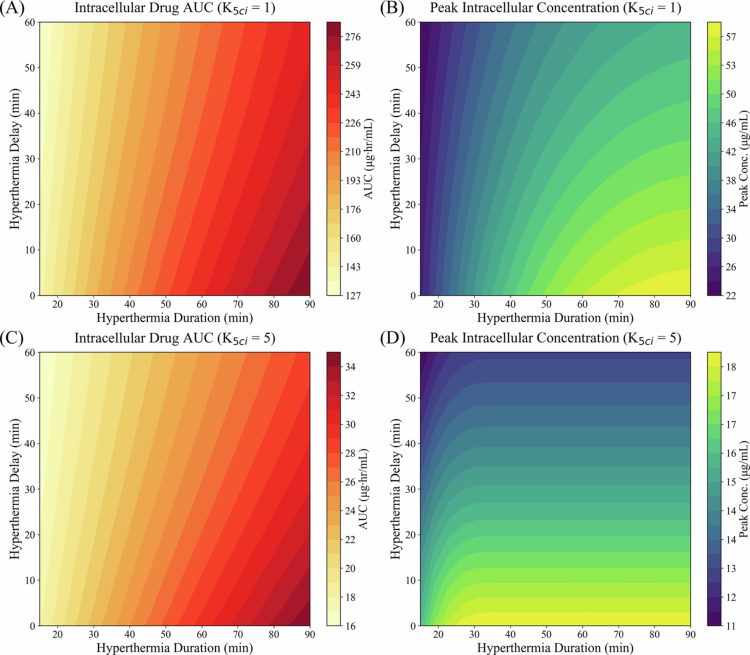
Dependence of intracellular DOX AUC (A, C) and $C_{i,\;\max}$ (B, D) on the combined effects of hyperthermia timing and duration for NSCLC uptake kinetics at different levels of cellular efflux. Panels (A) and (B) correspond to baseline efflux activity ($K_{5\mathrm{ci}}=1$), while panels (C) and (D) represent elevated efflux activity ($K_{5\mathrm{ci}}=5$).

Figure 7 illustrates the combined effects of hyperthermia duration and initiation delay on intracellular DOX exposure (AUC) and $C_{i,\;\max}$ under different levels of cellular efflux activity in NSCLC. For low efflux conditions ($K_{5\mathrm{ci}}=1$), both AUC and peak concentration increased with hyperthermia duration but decreased with delayed hyperthermia initiation. Notably, $C_{i,\;\max}$ exhibited saturation behavior at longer heating durations, whereas AUC continued to increase more gradually. Under elevated efflux conditions ($K_{5\mathrm{ci}}=5$), representing increased drug resistance, these suppressive effects were amplified.

### Impact of hyperthermia schedules on systemic free DOX exposure

**Figure 8.**
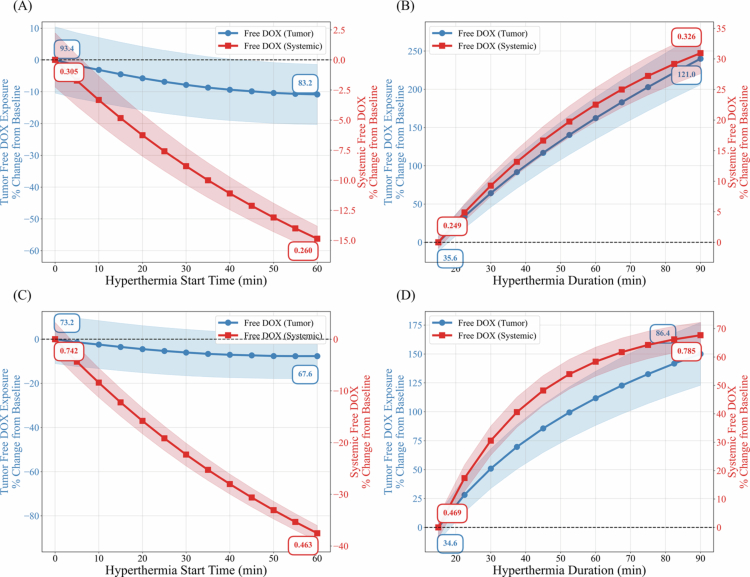
Changes in tumor and systemic plasma exposure to free DOX under different hyperthermia schedules, including variations in hyperthermia initiation time relative to peak TSL-DOX plasma concentration (A and C) and heating duration (B and D). Panels (A) and (B) correspond to the baseline tumor size, while panels (C) and (D) represent a 10-fold larger tumor. Absolute values are reported in μg⋅h/mL.

Figure 8 summarizes the percent change in free DOX exposure in tumor and systemic compartments relative to the baseline hyperthermia schedule. For the baseline tumor size (Figure 8A), delaying hyperthermia initiation from 0 to 60 min resulted in a progressive reduction in tumor uptake of about 10%, accompanied by a 15% reduction in systemic free DOX exposure. In contrast, prolonging hyperthermia duration from 15 to 90 min (Figure 8B) increased tumor uptake by up to 240%, while systemic free DOX exposure increased by about 30%.

For the larger tumor size (10-fold increase; Figure 8C and D), similar qualitative trends were predicted. Delayed hyperthermia initiation reduced tumor uptake by about 10% and systemic free DOX exposure by up to 40% (Figure 8C). Prolonged hyperthermia increased tumor uptake to about 150% above baseline, while systemic free DOX exposure increased by about 70% (Figure 8D). Although tumor uptake remained higher than systemic exposure across schedules, the magnitude of tumor-to-systemic selectivity gain was less pronounced for the larger tumor size.

### Comparison of continuous vs. fractional hyperthermia

**Figure 9.**
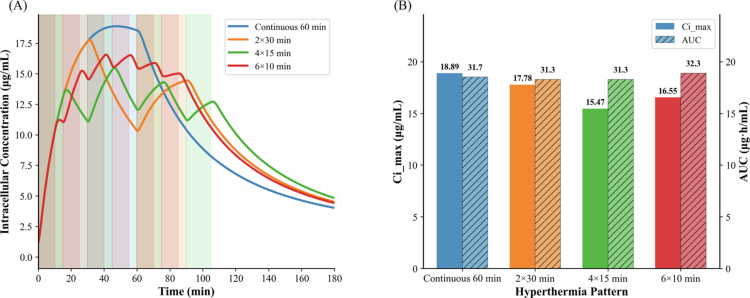
Comparison of continuous and fractional hyperthermia, each with a total duration of 60 min. (A) Intracellular concentration profiles. (B) $C_{i,\;\max}$ and corresponding AUC for different thermal delivery patterns. $K_{5\mathrm{ci}}$ was set at mid-range value of 5.

Figure 9 compares the intracellular concentrations resulting from continuous and fractional hyperthermia, each with a total duration of 60 min, based on uptake in L-DAN (NSCLC). Figure 9A shows the intracellular DOX concentration profiles, while Figure 9B presents the corresponding $C_{i,\;\max}$ and AUC values. Model predictions indicate that continuous hyperthermia achieves a higher peak intracellular concentration ($C_{i,\;\max}$) than fractional schedules. For example, $C_{i,\;\max}$ was about 20% higher with continuous 60-min heating compared to four 15-min fractions with 15-min cool-down intervals, while the corresponding AUC values were nearly identical.

## Discussion

This study used mathematical modeling to examine how hyperthermia timing, duration, and heating pattern influence TSL-i-mediated DOX delivery, as determined by cellular uptake in breast cancer and NSCLC. The physiological parameters of the model were based on available mouse data. The maximum intracellular concentration was considered the primary index of therapeutic efficacy, as preclinical data indicate a strong correlation between intracellular DOX levels and cell kill. Our findings suggest that hyperthermia should be initiated as early as possible when liposomal DOX is at peak systemic concentration, since delays in initiating hyperthermia reduce efficacy. Extending the duration of hyperthermia improved intracellular DOX concentrations, although the benefit diminished beyond 60 min in tumor cells with moderate to strong MDR phenotypes. Model predictions further indicate that prolonged hyperthermia enhances tumor uptake to a greater extent than systemic free DOX exposure, although this preferential enhancement is dependent on tumor size. Continuous hyperthermia produced higher intratumoral DOX levels compared with fractional hyperthermia.

Following administration, the concentration of TSLs in the bloodstream rises to a peak and then decays exponentially due to systemic elimination and drug release. Our results (Figure 5) show that delaying the onset of hyperthermia by 60 min significantly reduces the $C_{i,\;\max}$, with reductions of up to 25% or more depending on the cancer cell type. Given the 30-min infusion time for TSL-DOX in patients (Spiers et al. 2023), an appropriate protocol may involve 30 min of preheating to allow the tumor to reach the target steady-state hyperthermia temperature (42 °C–43 °C) (Ademaj et al. 2022), followed by 60 min of steady-state hyperthermia to maximize DOX uptake. In mice, our model predicts that applying hyperthermia within 40 min of the TSL-DOX plasma peak preserves about 80% of $C_{i,\;\max}$ for tumors following the DOX uptake kinetics of NSCLC and ER+/HER2− breast cancer, and within 20 min for triple-negative breast cancer (Figure 5D–F). To capture DOX uptake across tumor types, the model was calibrated with literature data from the L-DAN cell line for NSCLC, MCF-7 for ER+/HER2− breast cancer, and MDA-468 for triple-negative breast cancer. A comparable optimal window could be identified for humans using the same approach, provided that the relevant pharmacokinetic parameters are known. In line with our mathematical modeling findings (Figure 5), a recent *in vivo* study (Sebeke et al. 2022) in a porcine model using MR-guided HIFU reported more than a twofold increase in DOX concentration in the target tissue when hyperthermia was applied 10 min after TSL-DOX administration, compared with application delayed to 60 min.

We further investigated the effect of hyperthermia duration on intracellular DOX concentrations, while also considering the MDR phenotype of tumor cells to capture their altered uptake characteristics. Although MDR can result from multiple mechanisms, it is often associated with increased efflux mediated by ATP-binding cassette transporters, which reduce intracellular retention and efficacy (Gottesman et al. 2002; McKenna et al. 2017; Garg et al. 2024). For example, MCF-7/Adr, an MDR variant of the MCF-7 cell line, shows reduced DOX uptake and higher viability under treatment (Miri et al. 2023). Likewise, MDA-MB-468_MDR1_, derived from the triple-negative MDA-MB-468 line, overexpresses MDR1 and displays reduced DOX accumulation with enhanced resistance (McKenna et al. 2019). According to the results (Figure 6), intracellular DOX levels in triple-negative breast cancer cells were more responsive to hyperthermia compared with ER+/HER2− breast cancer, while NSCLC exhibited lower concentrations due to differences in uptake kinetics. This implies that, under a standard 60-min hyperthermia protocol, dose selection may need to be tailored to each cancer type and subtype to achieve the desired cytotoxic level. MDR cells, however, showed markedly reduced intracellular concentrations under the same conditions relative to sensitive cells. In our model, MDR was represented by an elevated efflux rate, and prolonging hyperthermia did not fully compensate for the resulting loss in drug retention. Combination strategies, such as co-delivery of efflux pump inhibitors like Tariquidar with TSL-i chemotherapy, may therefore help overcome the limited benefit of extended hyperthermia in MDR tumors (McKenna et al. 2019; Rani et al. 2024).

For NSCLC specifically, our results show that the dependence of intracellular concentration on hyperthermia duration diminishes beyond 60 min for DOX-sensitive cells, and beyond 30 min for moderately resistant cells (Figure 6). An *in vivo* study (Motamarry et al. 2019) employed real-time fluorescence imaging to evaluate local DOX uptake in Lewis lung carcinoma tumors, a murine lung cancer model commonly used as a preclinical surrogate for human NSCLC, during hyperthermia-mediated TSL-i delivery. The Wilcoxon Sum-Rank Test (five animals per group) showed a *p*-value of 0.4034 for the difference in DOX concentration between 60 and 30 min of hyperthermia, whereas the *p*-value between 30 and 15 min was 0.0601. Consistent with the insights from our mathematical model, these findings suggest that the difference in drug uptake is more pronounced between shorter hyperthermia durations.

The combined effects of hyperthermia timing and duration on intracellular DOX exposure, analyzed using NSCLC cellular uptake kinetics, were highly nonlinear (Figure 7). Peak intracellular concentration exhibited saturation at prolonged heating times, while the impact of extending hyperthermia duration was greater when hyperthermia was initiated earlier relative to TSL administration. Although prolonged hyperthermia increases systemic exposure alongside tumor exposure, our model predicts that the relative enhancement in tumor exposure is substantially greater than that in systemic exposure for the baseline tumor volume (Figure 8). These findings suggest that prolonged hyperthermia favorably alters the tumor-to-systemic exposure balance; however, the balance between therapeutic benefit and systemic toxicity is influenced by tumor size and physiological scaling.

For an equal thermal dose, we compared continuous and fractional hyperthermia as triggers for drug release (Figure 9). Continuous hyperthermia achieved higher $C_{i,\;\max}$, resulting in greater intracellular DOX levels. These findings indicate that drug release depends not only on the total thermal dose but also on the synchronization of drug availability and heating and highlight the importance of early heat application. Fractional heating schedules with short heating cycles followed by long cool-down periods missed the critical window when TSL-DOX concentrations were highest in tumor plasma. Supporting our results, an *in vivo* mouse study (Santos et al. 2017) using focused ultrasound hyperthermia (10×30 s bursts with 5 min cool-down versus 3.5 min continuous heating) also reported lower drug signal for fractional hyperthermia compared to continuous heating. However, the relative advantages of continuous versus fractional hyperthermia might be context-dependent and influenced by clinical constraints such as safety, patient tolerance, and heating feasibility. Recent experimental work (Li et al., 2025) has shown that fractional (cyclic) hyperthermia can improve the spatial distribution of DOX within tumors, whereas continuous hyperthermia yielded higher overall intratumoral drug accumulation, with comparable delayed tumor growth observed for both schedules. Fractional hyperthermia may therefore represent a clinically relevant alternative in scenarios where prolonged continuous heating is limited by more risk of tissue damage, vascular compromise, or technical constraints.

Although the present work focuses on TSL-mediated delivery of DOX, the proposed mathematical framework is not inherently drug-specific. In principle, the model could be extended to other chemotherapeutic agents by modifying a limited set of drug-dependent parameters. However, recent reviews (Haemmerich et al. 2023; Alhussan et al. 2025) and clinical studies (Thermosome GmbH 2025) indicate that DOX remains the most reliable and extensively characterized payload for TSLs, owing to its high loading efficiency, sharp heat-triggered release, and clinical evaluation (e.g. LTLD/ThermoDox, THE001). In contrast, many alternative agents remain limited by formulation stability challenges and a lack of quantitative intravascular release data.

The present study systematically evaluated the influence of hyperthermia scheduling on drug release and delivery under controlled, model-based conditions. This computational framework is intended to guide future experimental and *in vivo* studies by identifying key parameters and trends that can be validated and refined through subsequent experimental work. While the current model effectively describes overall drug behavior using ordinary differential equations (ODEs), a limitation is its inability to capture spatial heterogeneity of physiological parameters, such as vascular distribution and permeability variations within the tumor. Temperature effects are incorporated through a release-rate function triggered at hyperthermic temperatures, under the assumption that the tumor has reached a uniform steady-state temperature. To evaluate the thermal assumptions underlying the compartmental model, a spatially resolved bioheat simulation based on the Pennes equation was performed (Supplementary Figures S1 and S2). A representative simulation (Figure S1) illustrates the spatiotemporal temperature evolution under heterogeneous perfusion and specific absorption rate (SAR) fields, while an ensemble of N=30 simulations (Figure S2) quantifies the impact of spatial variability across different configurations of these fields. Tumor perfusion was modeled as a heterogeneous field spanning the physiological range reported in the literature (Mankoff et al. 2002), including both vessel-rich regions and low-perfusion necrotic-like zones. Spatially varying SAR fields were applied to reflect realistic heating non-uniformities. Intra-tumor temperature variability was on the order of ~$1.1{\;}^{\circ }C$ (standard deviation), driven by spatial heterogeneity in perfusion and energy deposition. These variations may lead to local differences in temperature-dependent drug release that are not captured in the current model. Despite this heterogeneity, the spatially averaged tumor temperature remains within the hyperthermic range ($42^{\;\circ }C-43^{\;\circ }C$). At steady state (*t* = 30 min), approximately 80% of the tumor area exceeds $42{\;}^{\circ }C$, indicating that therapeutically relevant conditions are achieved across a substantial fraction of the tumor. This assumption is further supported by invasive measurements in deep-seated breast tumors, which demonstrated relatively homogeneous intratumoral temperatures at steady state under 70 MHz waveguide heating (Crezee et al. 2017). For tumors with characteristic dimensions in the millimeter range, as considered in the present study, thermal diffusion and perfusion further reduce spatial temperature gradients. Under such conditions, once the target temperature range is achieved and maintained, drug release kinetics are governed primarily by temperature magnitude and exposure duration. Accordingly, tissues are represented as interconnected compartments, and model outputs reflect spatially averaged drug concentrations.

The validated computational framework provides a mechanistic basis that could, in future work, be coupled to spatial bioheat and imaging-derived models. A key next step toward this goal is extending the model to account for spatial thermal gradients and heterogeneous physiological properties, including voxel-level variability in permeability-surface area product and perfusion. In this setting, three-dimensional temperature maps derived from thermometry could be incorporated as reference fields to drive spatially resolved release kinetics across the tumor volume. Interpatient variability in physiological parameters plays a critical role in intratumoral drug distribution and can limit uniform drug exposure across tumors. For instance, in well-vascularized tumors, equilibrium between tumor plasma and EES is reached more rapidly, leading to higher intracellular drug concentrations. In tumors with short vascular transit times, incomplete intravascular release may reduce drug accumulation. To address such interpatient variability, the physiological parameters in the model should be customized to individual patients. These parameters including compartmental volumes, volume transfer constant $K_{\mathrm{trans}}$ (to estimate the permeability-surface area product of the tumor), tumor volume fractions, and perfusion maps could be derived from DCE-MRI sequences. This way, patient-specific models can be developed and used to maximize intracellular drug concentrations while keeping free DOX levels in systemic circulation below the tolerance threshold. We believe this work provides valuable insights into the impact of hyperthermia schedules on the TSL-i drug delivery system and will guide future efforts to enhance TSL-based drug delivery for patients.

## Conclusion

This study calibrated and validated a numerical model to predict and analyze the distribution of DOX encapsulated in TSLs, and investigated the effects of hyperthermia duration, pattern, and timing on intracellular drug concentrations in TSL-i DOX delivery. Optimal drug delivery was achieved when hyperthermia within the maximum release temperature range (42 °C–43 °C) was applied as soon as TSLs reached their peak concentration in systemic plasma. Prolonging hyperthermia increased intracellular drug accumulation, with a larger effect in DOX-sensitive cancer cells, whereas no significant additional benefit was predicted beyond 60 min in highly MDR NSCLC and ER+/HER2− breast cancer with elevated efflux activity. The enhancement in intratumoral drug accumulation exceeded the increase in systemic free DOX exposure under prolonged hyperthermia. Continuous hyperthermia, assuming an equivalent thermal dose, outperformed fractional hyperthermia in terms of peak intracellular concentration. Overall, this modeling approach and study provide key insights into optimizing hyperthermia-mediated TSL-i drug delivery and clarifies schedule-dependent delivery mechanisms under controlled hyperthermic conditions.

## Supplementary Material

Supplementary Material pNamakshenas_Supplementary.pdf

## Data Availability

Data supporting this study will be made available upon reasonable request from the corresponding author.

## Nomenclature

AUC: area under the curve
CF: carboxyfluorescein
DOX: doxorubicin
DPPG_2_: 1,2-dipalmitoyl-sn-glycero-3-phosphoglycerol
EES: extravascular extracellular space
EPR: enhanced permeability and retention
ER+: estrogen receptor positive
HER2: human epidermal growth factor receptor 2
MDR: multidrug resistance
MRI: magnetic resonance imaging
DCE-MRI: dynamic contrast-enhanced MRI
NSCLC: non-small cell lung cancer
ODE: ordinary differential equation
PEG: polyethylene glycol
TSL: thermosensitive liposome
TSL-DOX: thermosensitive liposomal doxorubicin
TSL-e: extravascular triggered release thermosensitive liposome
TSL-i: intravascular triggered release thermosensitive liposome
SAR: specific absorption rate
