# Supplementary material for: Exploiting the dynamics of hyperthermia-enhanced delivery of thermosensitive liposomal doxorubicin to solid tumors
Source: Drug Deliv. 2026 May 10;33(1):2670077. doi: 10.1080/10717544.2026.2670077 (PMC13162552; doi:10.1080/10717544.2026.2670077)
Supplement: Supplementary Material — pNamakshenas_Supplementary.pdf [file IDRD_A_2670077_SM1214.pdf]

Supplementary Material: Thermal Modeling under Heterogeneous Perfusion and Energy Deposition

Localized hyperthermia is inherently spatially heterogeneous: blood perfusion varies across vessel-rich and necrotic subregions, and electromagnetic energy deposition exhibits focal non-uniformities that are difficult to control clinically. This section evaluates whether such spatial heterogeneity compromises the assumption of thermal uniformity underlying the compartmental model used in the main text. To this end, we performed spatially resolved finite-difference simulations of the Pennes bioheat equation in a two-dimensional tissue domain. A reference simulation, together with a Monte Carlo ensemble ($N = 30$), was used to characterise the resulting intra-tumoral temperature distributions under imposed perfusion and SAR heterogeneity.

Pennes Bioheat Equation

Spatial temperature evolution was modeled using the two-dimensional Pennes bioheat equation [1]:

$$\rho_t\, c_t\, \frac{\partial T}{\partial t} \;=\; k_t\, \nabla^2 T \;+\; \underbrace{\omega_b(\mathbf{r})\, c_b \big[T_b - T(\mathbf{r},t)\big]}_{\text{blood perfusion cooling}} \;+\; \underbrace{Q_{\mathrm{SAR}}(\mathbf{r})}_{\text{heat source}} \tag{1}$$

where $T(\mathbf{r},t)$ is the local tissue temperature (°C), $T_b = 37\,°\mathrm{C}$ is the arterial blood temperature, $\omega_b(\mathbf{r})$ is the spatially heterogeneous blood perfusion rate ($\mathrm{kg\,m^{-3}\,s^{-1}}$), and $Q_{\mathrm{SAR}}(\mathbf{r})$ is the specific absorption rate (SAR, $\mathrm{W\,m^{-3}}$). The domain was a $8\,\mathrm{cm} \times 8\,\mathrm{cm}$ square grid ($N = 160$ nodes per axis, $\Delta x = 0.5\,\mathrm{mm}$) solved by an explicit finite-difference scheme with Dirichlet boundary conditions ($T_{\partial\Omega} = T_b$) and a time step $\Delta t = \min\big(0.25,\ 0.95\,\Delta x^2/4\alpha\big)$ s, where $\alpha = k_t/(\rho_t c_t)$ is the thermal diffusivity. Simulation ran for 30 min of physiological time.

Model Parameters

Heterogeneous Perfusion and SAR Fields

Tumour blood perfusion heterogeneity was introduced by superimposing six spatially localized Gaussian perfusion enhancements (peak ω_b up to $11.8\,\mathrm{kg\,m^{-3}\,s^{-1}}$) and one low-perfusion (necrotic) region (floor $\omega_b \approx 1.6\,\mathrm{kg\,m^{-3}\,s^{-1}}$) onto a uniform tumour background, with the spatial mean subsequently anchored to the Mankoff PET-derived value ($\omega_{\mathrm{tum}} = 5.88\,\mathrm{kg\,m^{-3}\,s^{-1}}$) [4].
The SAR field was constructed as a Gaussian distribution centred on the tumour ($\sigma_{\mathrm{SAR}} = 8\,\mathrm{mm}$) with six stochastic scatter blobs (amplitude ±30–38% of the target SAR), resulting in a spatial coefficient of variation CoV(SAR) of approximately 25 % (panel B of Fig. S1). Normal-tissue SAR was attenuated to 12 % of the intra-tumour value.

Monte Carlo Ensemble Analysis

To quantify the impact of spatial heterogeneity in perfusion and SAR, a Monte Carlo ensemble of $N = 30$ independent simulations was performed (Fig. S2). For each run, spatial variations in perfusion (local high- and low-perfusion regions) and SAR distribution were generated using independent random seeds. To isolate the effect of spatial heterogeneity, the tumour-averaged perfusion and SAR were kept constant across simulations. Consequently, differences in thermal outcomes arise from spatial variability rather than changes in mean energy input. For each simulation, temperature statistics were evaluated at $t = 30\,\mathrm{min}$ over tumour voxels, including: T_{10} (hottest 10%), T_{50} (median), T_{90} (coolest 10%), the thermal heterogeneity index (THI $= T_{10} - T_{90}$), and the percentage of tumour voxels exceeding 42 °C.

Simulation Results

The reference bioheat simulation demonstrated progressive heating of the tumour over the 30-minute hyperthermia period, with intra-tumoral temperatures stabilising at approximately $t = 15\,\mathrm{min}$ (Fig. S1C–F).

Table S1. Physical and numerical parameters used in the Pennes bioheat simulation.

Symbol	Parameter	Value	Unit	Ref/note
Tissue thermophysics				
ρ_t	Tissue density	1050	$\mathrm{kg\,m^{-3}}$	[2]
c_t	Tissue specific heat	3600	$\mathrm{J\,kg^{-1}\,K^{-1}}$	[2]
k_t	Thermal conductivity	0.50	$\mathrm{W\,m^{-1}\,K^{-1}}$	[2]
c_b	Blood specific heat	3651	$\mathrm{J\,kg^{-1}\,K^{-1}}$	[3]
T_b	Arterial blood temperature	37.0	°C	Normothermic
Blood perfusion (Mankoff et al. 2002 [4], ^{15}O-PET, $n = 37$)				
F_{tum}	Tumour mean perfusion	0.32	$\mathrm{mL\,min^{-1}\,g^{-1}}$	[4]
F_{low}	Necrotic region perfusion	0.15	$\mathrm{mL\,min^{-1}\,g^{-1}}$	[4]
F_{high}	Highly vascularised region perfusion	0.64	$\mathrm{mL\,min^{-1}\,g^{-1}}$	[4]
F_{norm}	Normal breast perfusion	0.06	$\mathrm{mL\,min^{-1}\,g^{-1}}$	[4]
ω_{tum}	Tumour ω_b (mean)	5.88	$\mathrm{kg\,m^{-3}\,s^{-1}}$	F_{tum}, converted[a]
ω_{norm}	Normal tissue ω_b	1.10	$\mathrm{kg\,m^{-3}\,s^{-1}}$	F_{norm}, converted[a]
SAR field				
σ_{SAR}	Gaussian lobe width	8.0	mm	EM focal width
N_{scatter}	Stochastic scatter blobs	6	—	±30–38% amplitude
CoV(SAR)	SAR coefficient of variation	25	%	Computed
Simulation domain and numerics				
L	Domain side length	80	mm	Square domain
N	Grid points per axis	160	—	$\Delta x = 0.5\,\mathrm{mm}$
R_{tum}	Tumour radius	10	mm	Spherical approximation
t_{end}	Simulation duration	1800	s	30 min hyperthermia
T_{target}	Target tumour temperature	43.0	°C	Therapeutic window

[a] Conversion: $\omega_b\ [\mathrm{kg\,m^{-3}\,s^{-1}}] = F\ [\mathrm{mL\,min^{-1}\,g^{-1}}] \times \rho_b \times 10^{-3}/60$, where $\rho_b = 1050\,\mathrm{kg\,m^{-3}}$.

At steady state, the tumour median temperature (T_{50}) reached 43 °C, with a spatial standard deviation of $\sigma_T \approx 1.1$ °C, driven by heterogeneous perfusion and SAR distributions. The 42 °C therapeutic isotherm encompassed the majority of the tumour area, although localised cold spots persisted in high-perfusion subregions (Fig. S1). The Monte Carlo ensemble ($N = 30$) confirmed that these thermal outcomes are robust to stochastic variation in spatial SAR and perfusion patterns (Fig. S2). Across simulations, the thermal heterogeneity index (THI $= T_{10} - T_{90}$) averaged 2.80 °C ($\sigma = 0.25$ °C). On average, $80.9, \%$ of tumour voxels exceeded the 42 °C threshold (range: 77.6–88.5,%), indicating that adequate tumour coverage is maintained despite stochastic heterogeneity in energy deposition and tissue vascularity.

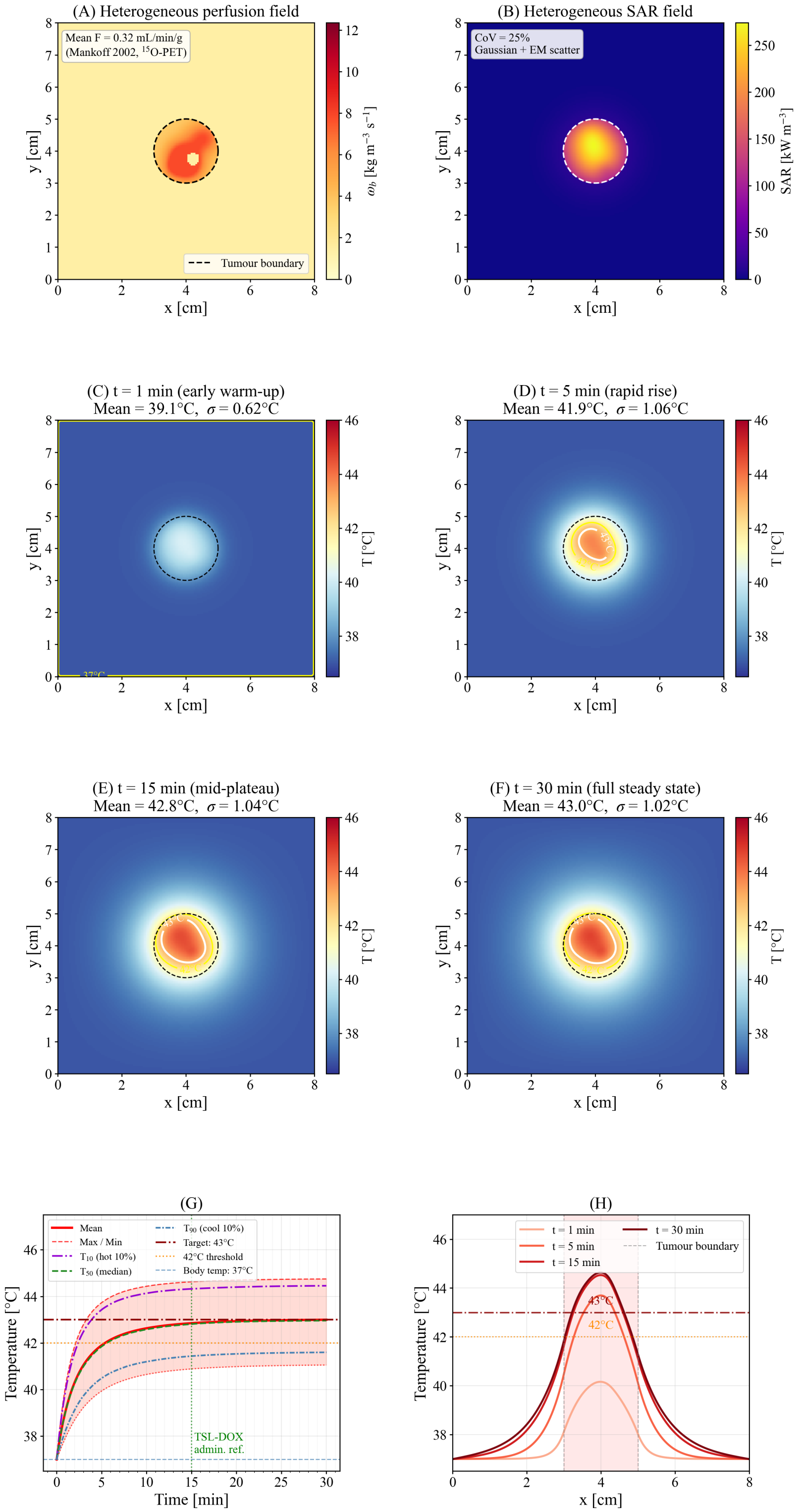

Fig S1. Spatiotemporal Pennes bioheat simulation of tumour hyperthermia (reference simulation). (A) Heterogeneous blood perfusion field $\omega_b(\mathbf{r})$; the dashed circle denotes the tumour boundary ($R = 10\,\text{mm}$). (B) SAR distribution ($\sigma = 8\,\text{mm}$) with stochastic scatter (CoV $= 25\%$). (C–F) Temperature maps at $t = 1$, 5, 15, and 30 min. Iso-contours indicate the 42 °C and 43 °C therapeutic thresholds. Intra-tumour temperature variability stabilises at $\sigma_T \approx 1.1\,°\text{C}$. (G) Temporal evolution of tumour temperature statistics: mean, min/max envelope, and $T_{10}/T_{50}/T_{90}$ percentiles. (H) Mid-plane temperature profiles ($y = 40\,\text{mm}$) at the four time points; the shaded region indicates the tumour extent.

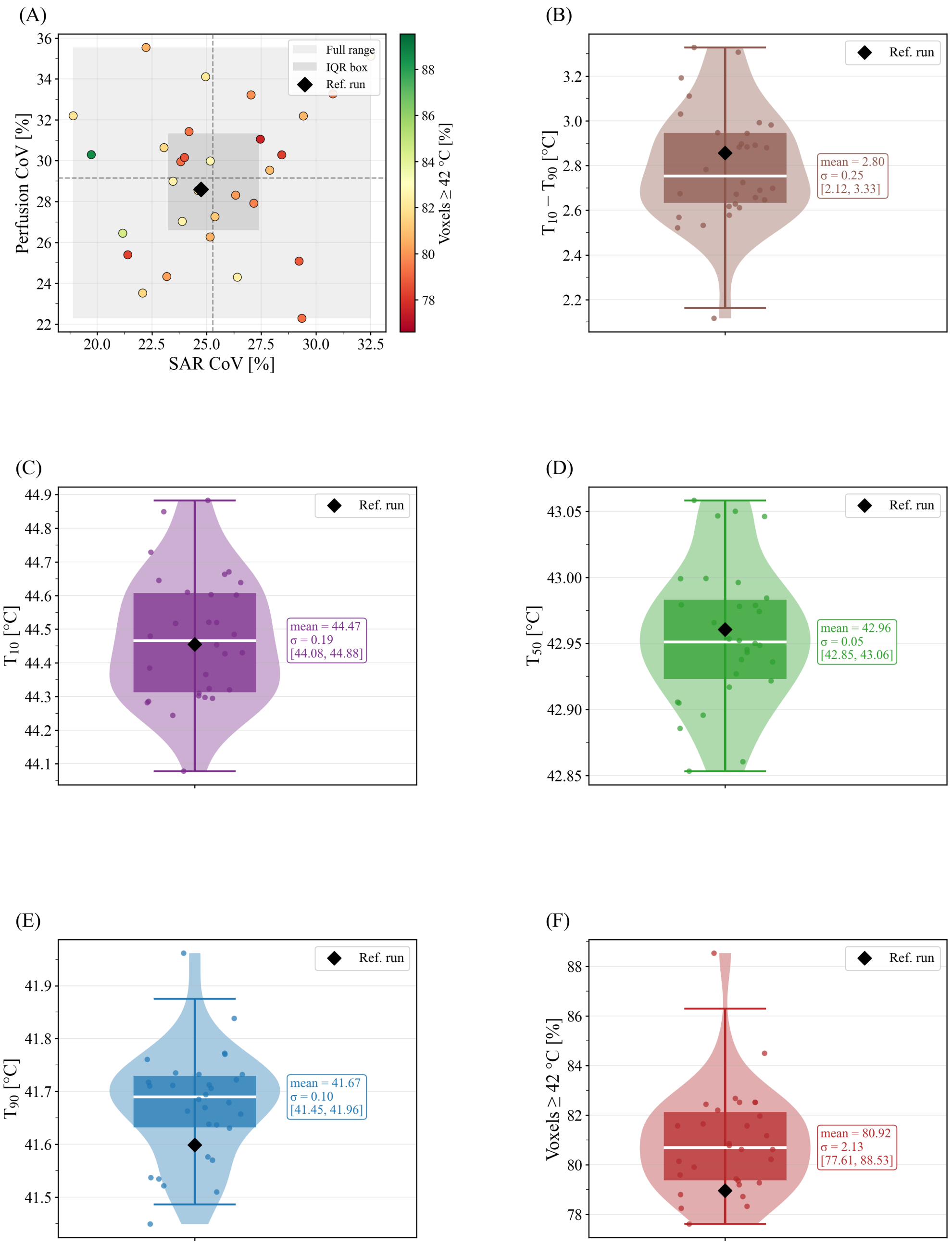

Fig S2. Ensemble sensitivity analysis of intra-tumoral hyperthermia metrics across stochastic SAR and perfusion maps ($N = 30$ simulations). (A) Joint distribution of SAR CoV and perfusion CoV, coloured by the percentage of tumour voxels $\geq 42\,°\mathrm{C}$. The black diamond denotes the reference run shown in Fig. S1. (B) Thermal heterogeneity index ($T_{10} - T_{90}$; mean $= 2.80\,°\mathrm{C}$, $\sigma = 0.25\,°\mathrm{C}$). (C–E) Steady-state distributions of the hot-spot (T_{10}; mean $= 44.47\,°\mathrm{C}$), median (T_{50}; mean $= 42.96\,°\mathrm{C}$), and cold-spot (T_{90}; mean $= 41.67\,°\mathrm{C}$) temperatures. (F) Distribution of tumour area fraction heated above $42\,°\mathrm{C}$ (mean $= 80.92\,\%$, $\sigma = 2.13\,\%$; range: 77.6–88.5 %). All metrics are evaluated at steady state ($t = 30\,\mathrm{min}$).

References

1. Pennes HH. Analysis of tissue and arterial blood temperatures in the resting human forearm. Journal of applied physiology. 1948;1(2):93-122.

2. Said Camilleri J, Farrugia L, Curto S, Rodrigues DB, Farina L, Caruana Dingli G, et al. Review of thermal and physiological properties of human breast tissue. Sensors. 2022;22(10):3894.

3. Mcintosh RL, Anderson V. A comprehensive tissue properties database provided for the thermal assessment of a human at rest. Biophysical Reviews and Letters. 2010;5(03):129-51.

4. Mankoff DA, Dunnwald LK, Gralow JR, Ellis GK, Charlop A, Lawton TJ, et al. Blood flow and metabolism in locally advanced breast cancer: relationship to response to therapy. Journal of Nuclear Medicine. 2002;43(4):500-9.
